# Water demand management: Visualising a public good

**DOI:** 10.1371/journal.pone.0234621

**Published:** 2020-06-16

**Authors:** Yurina Otaki, Hidehito Honda, Kazuhiro Ueda

**Affiliations:** 1 Hitotsubashi University, Kunitachi, Tokyo, Japan; 2 Yasuda Women’s University, Hiroshima Asaminami-ku, Hiroshima, Japan; 3 University of Tokyo, Meguro, Tokyo, Japan; Middlesex University, UNITED KINGDOM

## Abstract

Recent studies on water demand management show that providing visual information on water usage along with social comparisons with neighbouring households resulted in more efficient water usage. However, social comparisons can be discomforting for participants, especially in the case of downward or negative evaluations. To avoid this, some studies promote the use of social identity, a social norm approach that avoids comparisons. Past studies using social comparison used infographics, whereas other study types have used only textual (non-graphic) information. Therefore, in this study, we created a visualisation of water usage to highlight the importance of water as a shared resource, that is, as a public good, and feedback over six months according to the participants’ water usage. A difference-in-difference analysis indicated that the feedback was marginally significant in decreasing water consumption immediately and continuously, especially for the middle and low use households, during the summer months, which is a period of perceived water shortage. From the questionnaire survey, we found that households felt that they determined their water usage based on their preference and were satisfied with the outcome.

## 1. Introduction

The demand-side management of both energy and water has recently attracted much attention [1–3]. In particular, smart meters have facilitated the availability of detailed water usage data for users [4–7]. Many studies have examined the potential for encouraging energy- and water-saving behaviours by sharing the household’s data, as well as peer usage data. This strategy of providing usage feedback via social comparison is more effective in changing water usage behaviours than educational or awareness campaigns [8].

While providing social comparison feedback, some studies have relied on numerical information such as individual and average household usage levels, [3,9,10] as well as their rankings among the participants of field experiments conducted in a nearby region [9,11]. In terms of results, some of these studies reported a decrease in water usage [10,11], whereas others reported status quo usage [3,9]. Moreover, in contexts other than water and energy, some researchers reported that the provision of peer information only by text messaging leads to socially undesirable behaviour in the case of low performers (i.e. a study regarding savings reported that peer information via text led low-saving individuals to further decrease their savings [12], and another study regarding health reported that peer information sent via text led low-performers to use health equipment less [13]. Thus, to create more effective usage feedback interventions, attempts have been made to design visual feedback indicating water usage patterns of individual, average household, and efficient neighbouring household in the form of bar charts [2,11,14–16], emoticons [2,6,9,14–18], and circles whose size reflects the quantity consumed [19], as well as competitive framing [20]. Most of these studies reported a successful decrease in water usage by ‘high consumers’ and successful control of the boomerang effect (i.e. providing information in an attempt to reduce water usage increases water usage) for ‘low consumers’.

However, such social comparison feedback could be uncomfortable for participants, especially in the case of negative evaluations [21]. To avoid such a situation, some studies used a social norm intervention without comparisons: social identity. One’s social identity reflects an individual’s membership in a social group together with the value and emotional significance she/he attaches to it [22]. The more a person identifies with a group, the more likely her/his behaviour aligns with the group's norms [23], resulting in cooperation in the provision of public goods shared by the group [24]. A study in Cobb County, Georgia, used textual information encouraging the protection and conservation of ‘our’ environment and ‘our’ water resources and emphasize water as ‘our’ common good. This intervention was effective only for short-term water savings, as the effects failed to persist [25]. Another study targeting affluent households in Los Angeles also used textual information within a social identity approach to promote the need for water conservation and pro-environmental behaviour using the term ‘our’ city; however, ‘your’ was used in the simultaneous presentation of personal identity. The results of this study were similar to those using social comparison, as they indicated a reduction in water usage by high consumers in both the short and long term [18]. Overall, although previous studies using social comparisons used infographics, most studies that provided feedback information other than social comparisons used only textual information to emphasise the importance of environmental protection and water conservation for the common good. To fill this gap, this study proposes a visualisation of water usage information to highlight the importance of water as a resource shared by residents (that is, a public good) and observes the subsequent changes in consumption patterns.

## 2. Materials and methods

### 2.1. Overview

This study examines the efficacy of conceptualising water as a public good to encourage efficient water usage. First, a random sample of 170 households in Tokyo was drawn from a roster of survey registrants of a research company. The participants were randomly assigned into either a treatment group (hereafter, feedback group) that received feedback or into a control group that did not. Water consumption was monitored through water-meter readings and reported by the participants once every two weeks over twenty-four weeks from May 2018 to October 2018, resulting in twelve observations per participant. The first two times were used to determine the baseline consumption and the next ten were used to provide feedback information. Participants in the feedback group received e-mails informing them about their water consumption to stimulate greater public good awareness within a few days of each observation. Then, participants were given a questionnaire on how they had changed their water usage and how they felt about receiving feedback.

R version 3.6.2 was used to conduct all the statistical analyses and for drawing some figures, Microsoft excel 2016 was used for drawing some figures, and Illustrator 16.0 was used for creating the feedback visuals.

This study received approval from the Hitotsubashi University Research Ethics Committee, and participants’ consent was obtained electronically through the web-site.

### 2.2. Water resources in Tokyo

In Tokyo, water for residential use is stored in eleven dams. In normal years, the amount of available water is usually sufficient, but once every few years there is an acute shortage during the summer months (July to September) [26]. There was no shortage in 2018 when the survey was conducted, but the years 2001 (from 10 August to 27 August), 2012 (from 11 September to 3 October), 2013 (from 24 July to 18 September), and 2016 (from 16 June to 2 September) witnessed limited water resources.

**Fig 1:**
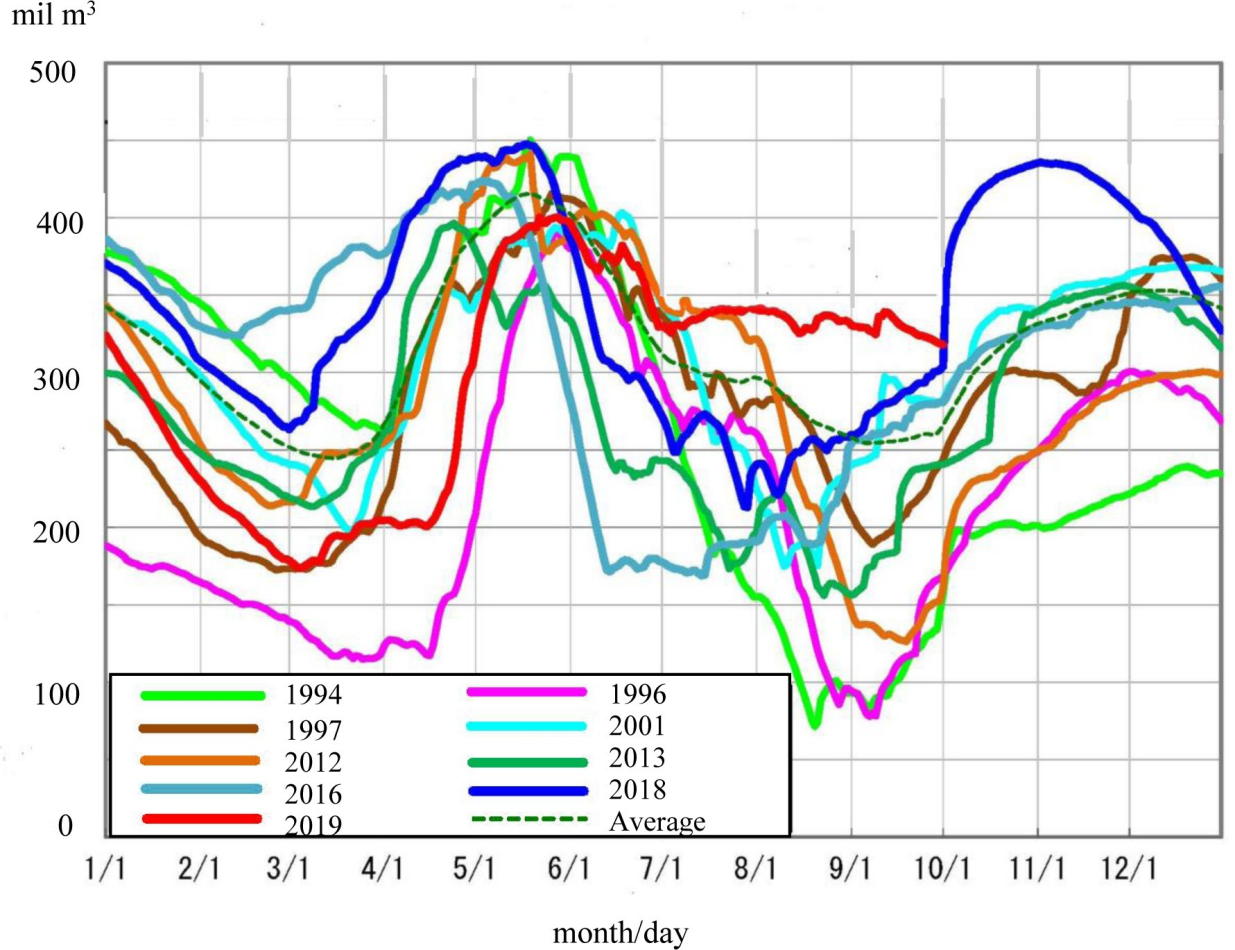
Water stored in the main dams in Tokyo.

**Fig 2:**
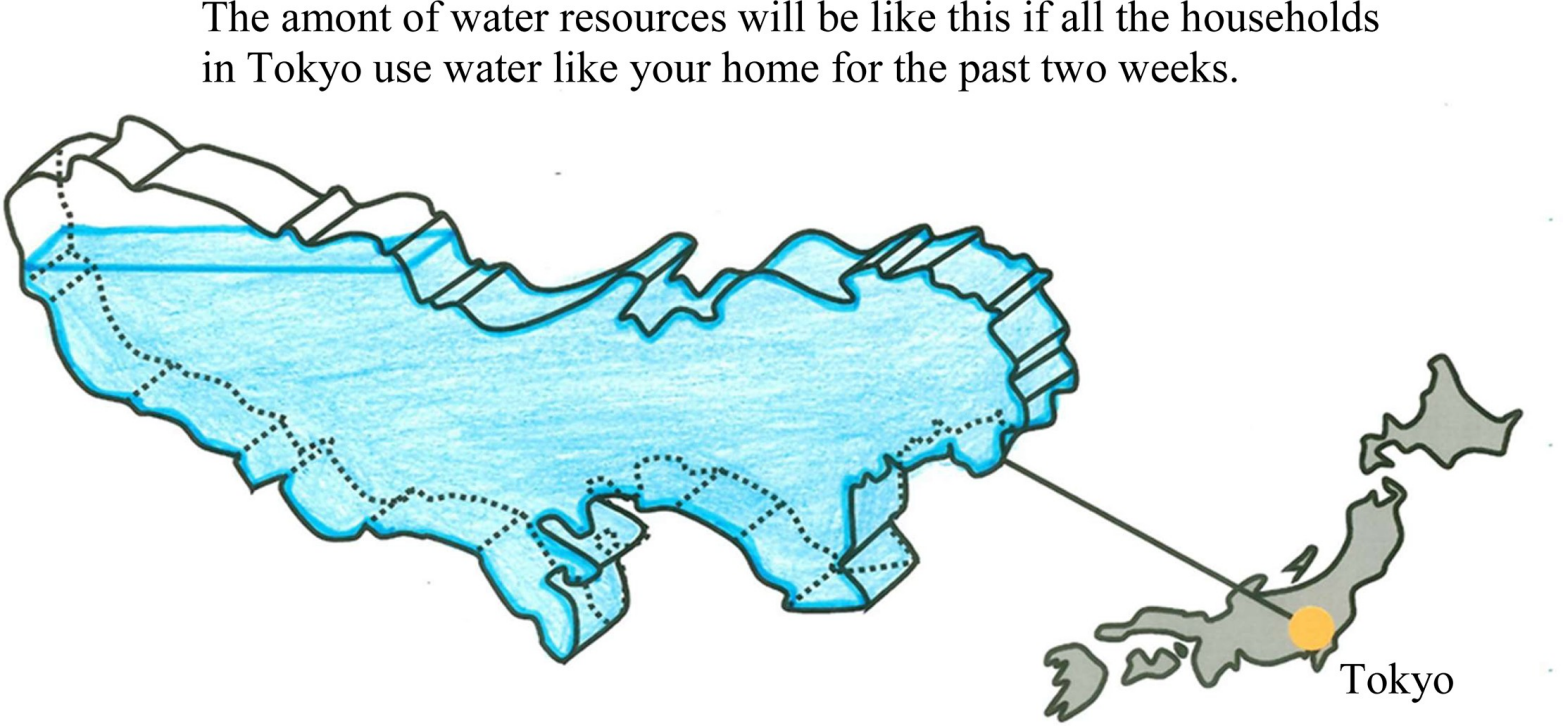
Example of visual feedback.

Fig 1 shows the amount of water resources stored in Tokyo’s main dams [27]. The amount of stored water generally decreases between July and September and increases in October. The media often report water shortages in the dams. Therefore, providing information on the amount of water in the dams was assumed to be the most appropriate way to generate awareness of water as a public good among residents in Tokyo. As shown in Fig 2, we visualised Tokyo as a vessel indicating water consumption of an individual household and then showed the amount of water in Tokyo’s main dams if all households continued to use the same amount of water as the individual household over two weeks. Before the start of the intervention, we explained to the participants that the shape of the vessel was in the form of Tokyo so that everyone could recognize it.

### 2.3. Intervention

As shown in Fig 2, participants in the feedback group received water vessel visuals via e-mail every two weeks from early June to mid-October, for a total of 10 times. The water level in the visual differed depending on individual consumption over the period. The lower the amount of water used, the higher the level of water in the visual vessel. We prepared eight patterns of water vessel visuals with different water levels (S1 Fig), and participants received the visuals depending on their usage during the prior two weeks. Daily per capita water consumption of each household was arranged in order of usage, divided into eight equal parts, and applied to each visual. As per capita water consumption is one of the important indicators in the context of water demand management [28,29], we used it for feedback and analysis.

### 2.4. Water consumption data and evaluation of changes

Per capita water consumption data were measured daily (l/capita/day) to smooth out differences across households due to household size heterogeneity. Water consumption becomes zero when the residents are away from home and becomes large when there are visitors to the home. To exclude conditions that are different from daily life from the analysis, observed values that were either extremely small or large were excluded using z values calculated from water consumption data collected during the survey period for each household. More specifically, we excluded observations with z-values greater than 2.5 or less than -2.5, that is, 1.8% of the data.

As the intervention began in June 2018, water consumption data in May were considered as the baseline. There was no statistically significant difference in the baseline consumption of the two groups (*t* (132) = -0.91, *p* = 0.37). The change in water consumption of each household was evaluated as follows:

LRPn=log(CnC5) (1)

where *C*_*5*_ is the monthly water consumption in May (baseline), *C*_*n*_ is the monthly water consumption after the intervention, and *LRP*_*n*_ is the log-transformed relative proportion (*n* = 6 for June, 7 for July, 8 for August, 9 for September, and 10 for October). A negative *LRP*_*n*_ indicated that water consumption had decreased, whereas a positive *LRP*_*n*_ indicated that it had increased.

First, we analysed water consumption changes over the time-series data using polynomial approximation. Subsequently, we ran a difference-in-differences (DID) approach for each month to determine how the visual feedback influenced water use behaviour using *LRP*_*n*_ at the household level as follows:

$${LRP}_{ni}=\mu +\gamma {Treatment}_{i}+\delta {Time}_{n}+\gamma {Treatment}_{i}\times {Time}_{n}+\varepsilon_{ni}$$ (2)

where *Treatment* is the dummy variable indicating whether households were in the feedback or control group, *Time*_*n*_ is the dummy variable indicating the time period in question, *i* is the household identifier, and *ε* is the error term.

**Fig 3:**
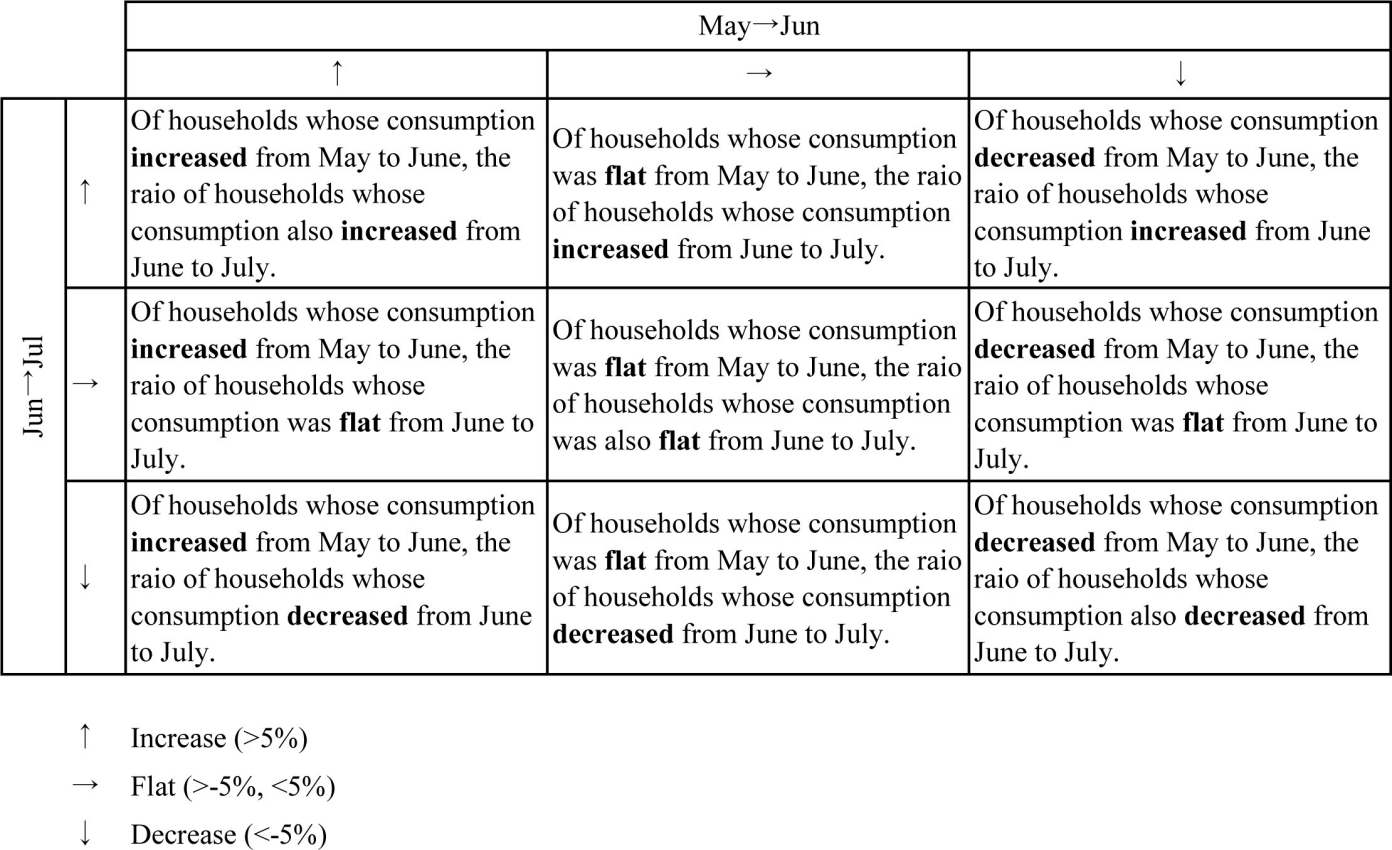
Month-to-month change matrix. ↑ increase (>5%); → flat (>-5%, <5%); ↓ decrease (<-5%).

Further, each household’s month-to-month water usage was mapped to analyse how the water usage change in the previous month related to that in the following month. As shown in Fig 3, the comparison with the previous month was categorized into three stages: increase (more than 5%), flat (more than -5% and less than 5%), and decrease (less than -5%).

### 2.5. Participants

**Fig 4:**
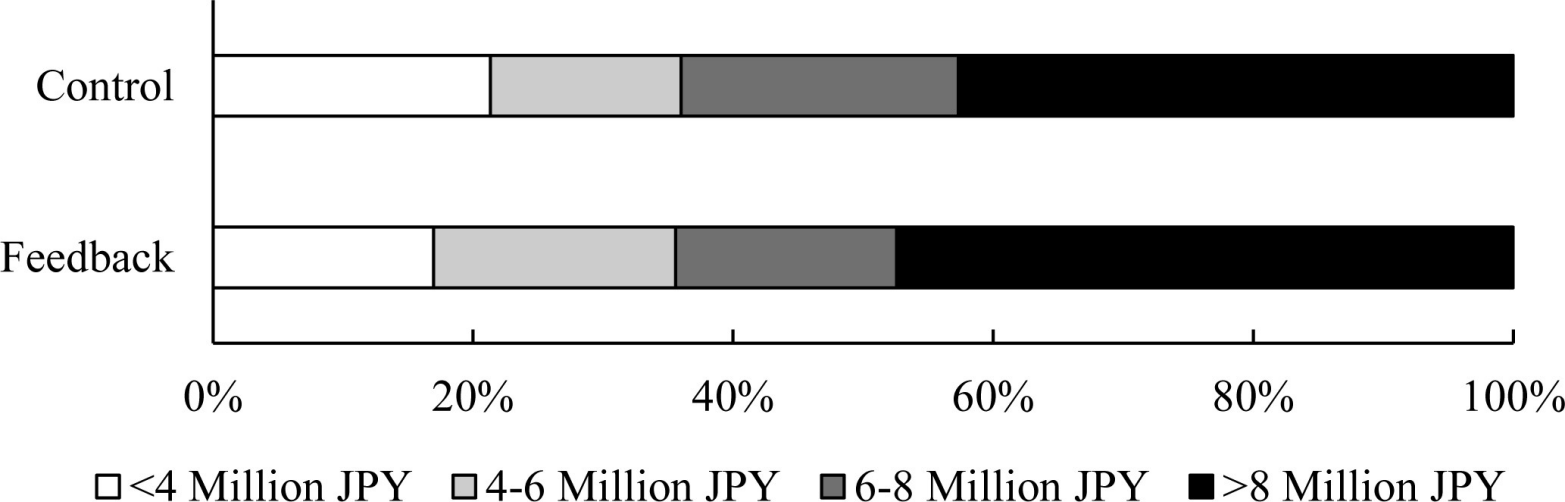
Distribution of annual household income.

This study was conducted among residents of Tokyo. Of the initial 170 households, 134 (75 in the control group and 59 in the feedback group) continued to participate (i.e. read the meter and received feedback) until the end of the study. The average number of members in a household was 2.65 (*SD* = 1.17), with 17%, 34%, 21%, 22%, and 5% of the households having one, two, three, four, and five or more members, respectively. S2 Table provides the distribution of the number of family members. There was no statistically significant difference among groups (*t* (132) = -0.25, *p* = 0.80). Fig 4 shows the distribution of annual household incomes, a chi-square test for its distribution indicated no evidence of a statistically significant difference between the control and feedback groups (*χ*^*2*^ (3) = 1.14, *p* = 0.77).

We classified the households in terms of the baseline water consumption. It is generally understood that as the number of household members increases, water consumption per capita decreases. To eliminate the influence of household size, we converted each household’s baseline water consumption per capita into a value that is assumed for a one-person household. In this way, each household was distributed into a ‘high’, ‘middle’, or ‘low’ level of water usage [30]. Low-use households are defined as those in the bottom third of water use, and high-use households are defined as those in the top third [20].

### 2.6. Questionnaire

At the end of the survey, a questionnaire was conducted to confirm the following three points. The first was whether the water usage was changed consciously or unconsciously. The second was whether individuals who were guided by socially desirable behaviour (water-saving behaviour in this study) felt that their free will was infringed. The third was whether these individuals felt any dissatisfaction with the feedback information. All participants were asked the following three questions corresponding to those three points, answered using a 10-point scale: (1) By how much has your water usage changed? (2) Did you determine your water usage based on your preferences? (3) Are you satisfied with your water usage? The full questionnaire is described in S2 File.

## 3. Results

### 3.1. Water consumption trends

**Fig 5:**
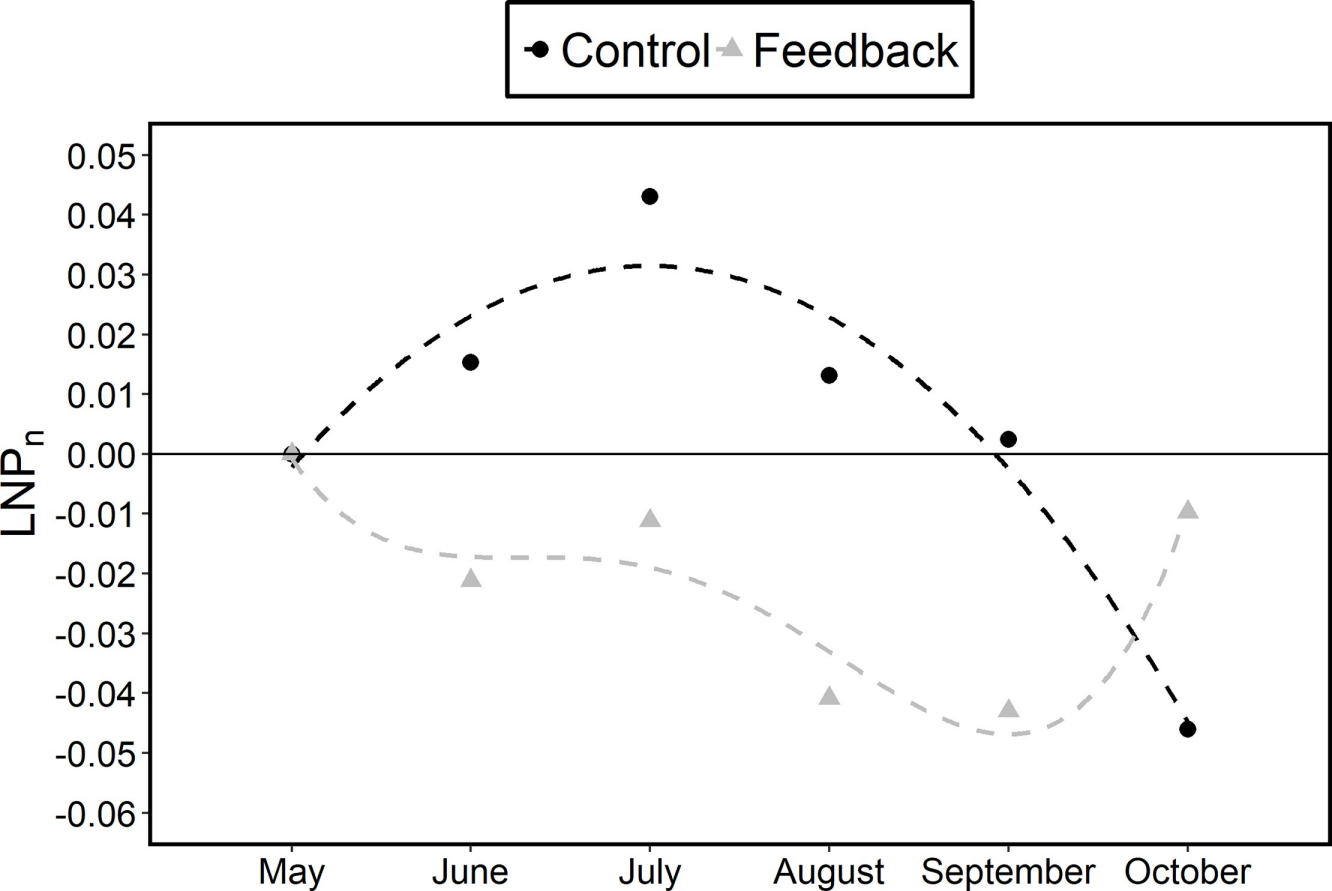
Change in the mean of *LRP*_*n*_ (n = 5–10) of each group over time.

Fig 5 shows, for each group, the change over time in the mean of *LRP*_*n*_ (n = 5–10). The mean value of the feedback group was consistently lower than that of the control group from June to September, but the differences disappeared in October. We fitted the polynomial approximation and calculated the AIC value for each group. The AIC value was lowest in the case of n = 2 for the control group and n = 4 for the feedback group. This finding means that the water consumption change of the control group was approximated by a quadratic curve and that of the feedback group was approximated by a fourth-dimensional curve. Because approximation polynomials differ depending on the group, we conducted a monthly DID analysis.

A difference-in-differences analysis indicated that the feedback was marginally significant to decrease water consumption from May to September (Table 1). The result of the analysis with excluding outlier judged by z-value (described in 2.4) is shown in S4 Table. Basically, the outlier did not change the result of the statistical analysis. Although the trend was the same, in September, the result slightly differed. This was because there was temporary increase in water consumption more than twice the normal value in the feedback households in September. Therefore, it could be presumed that the difference in September was not due to feedback but a factor peculiar to the relevant household.

**Table 1:** Result of difference-in-differences analysis.

Month	*t* value	*p* value
June	-1.808	0.072*
July	-1.907	0.058*
August	-1.784	0.076*
September	-1.701	0.090*
October	1.167	0.244

* indicates significant at the 10% level.

**Fig 6:**
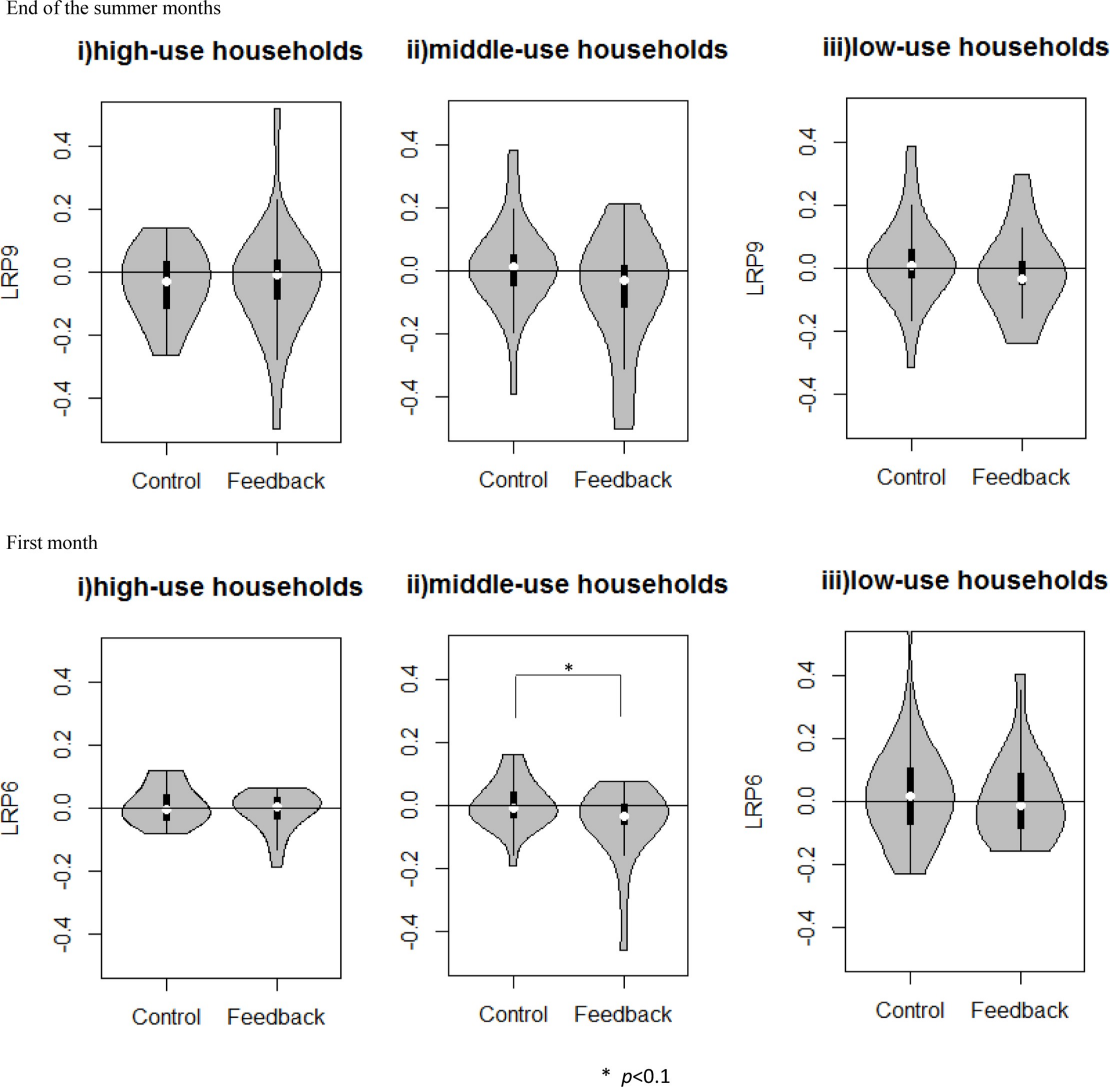
Feedback effect for the first month (LRP_6_) and the end of the summer months (LRP_9_) according to the three usage levels.

Fig 6 presents the feedback effect for the first month (LRP_6_) and the end of the summer months (LRP_9_) according to the three usage levels. In the first month, the feedback was marginally significant in decreasing middle-use households’ consumption (*W* = 335, *p* = 0.099). At the end of the summer months, the feedback tended to decrease the low water user’s consumption.

**Fig 7:**
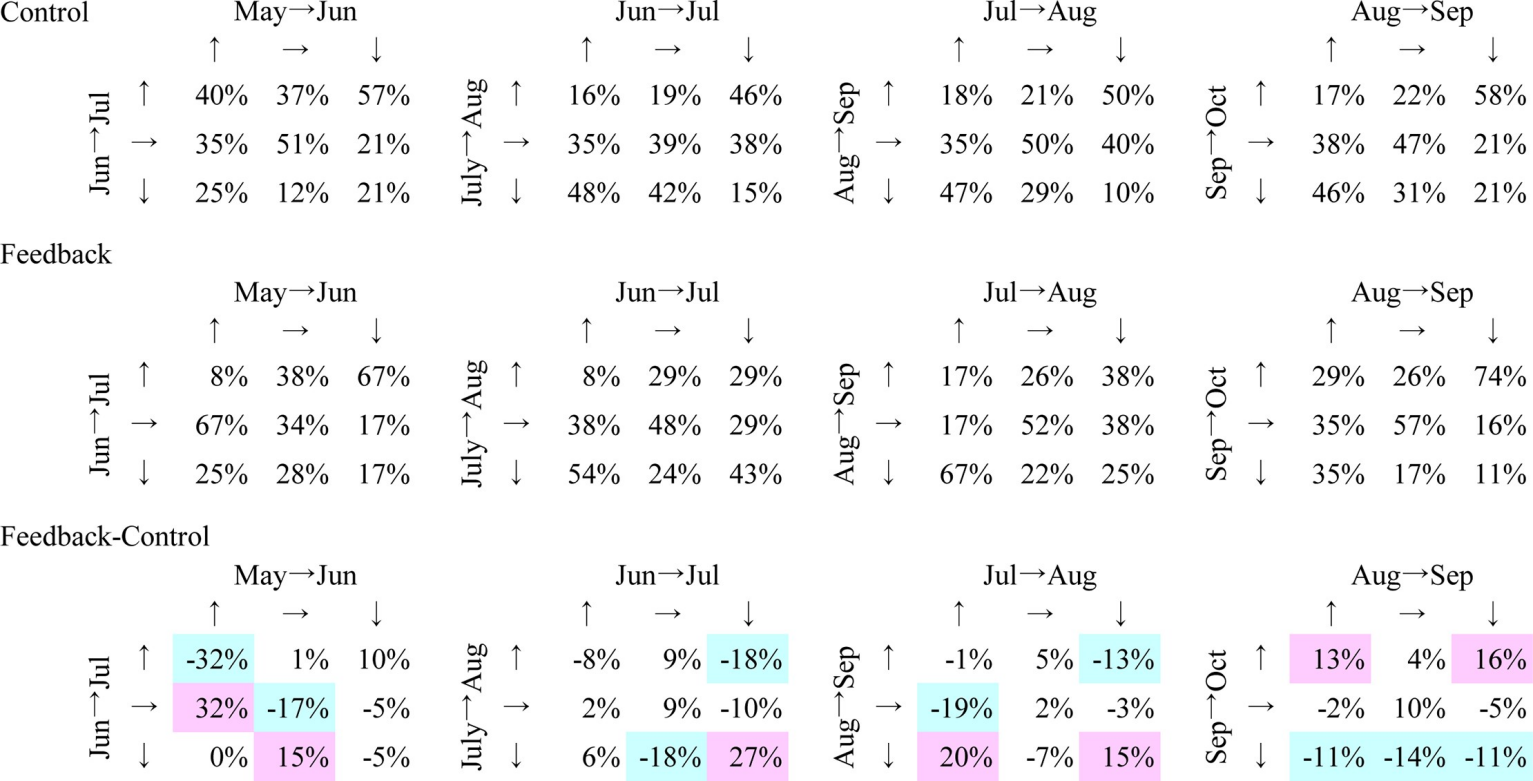
Month-to-month changes from May to October.

Fig 7 categorizes the month-to-month water consumption change. As explained in Fig 3, we described matrices of the consumption change rate from two months prior to the previous month and from the previous month to the current month for each group (control group at the top and feedback group in the middle), as well as their difference (control-feedback at the bottom). In the bottom matrices, the negative value indicates that the ratio of the feedback group is relatively low compared to that of the control group, and the positive value indicates that the ratio of the feedback group is relatively large to that of the control group, and vice versa. For example, among households of the control group who increased their consumption from May to June, 40% increased, 35% did not change, and 25% decreased their consumption from June to July (leftmost column of the leftmost matrices in Fig 7). Moreover, in the control group, a relatively large ratio of households increased its consumption both from May to June and June to July; in the feedback group, a relatively large ratio of households increased their consumption from May to June, and subsequently, decreased it from June to July. We coloured the cells with values less and more than 10%, respectively, in blue and pink. The leftmost matrices show that households whose usage increased in the first month also increased their usage in the following month without feedback, and the same can be said for households with flat usage. From the second to the leftmost matrices, of the households whose usage decreased from June to July, households whose usage increased in the following month were mainly in the control group, and households whose usage further decreased were mainly in the feedback group. Regardless of the previous month, the second to the rightmost matrices indicated that feedback caused a downward trend in the following month. Overall, providing feedback through visuals conceptualising water as a shared public good resulted in the immediate and continuous reduction in water consumption. However, the rightmost matrices showed that in the last month, feedback caused an upward trend regardless of the previous month’s trend.

### 3.2. Questionnaire

**Fig 8:**
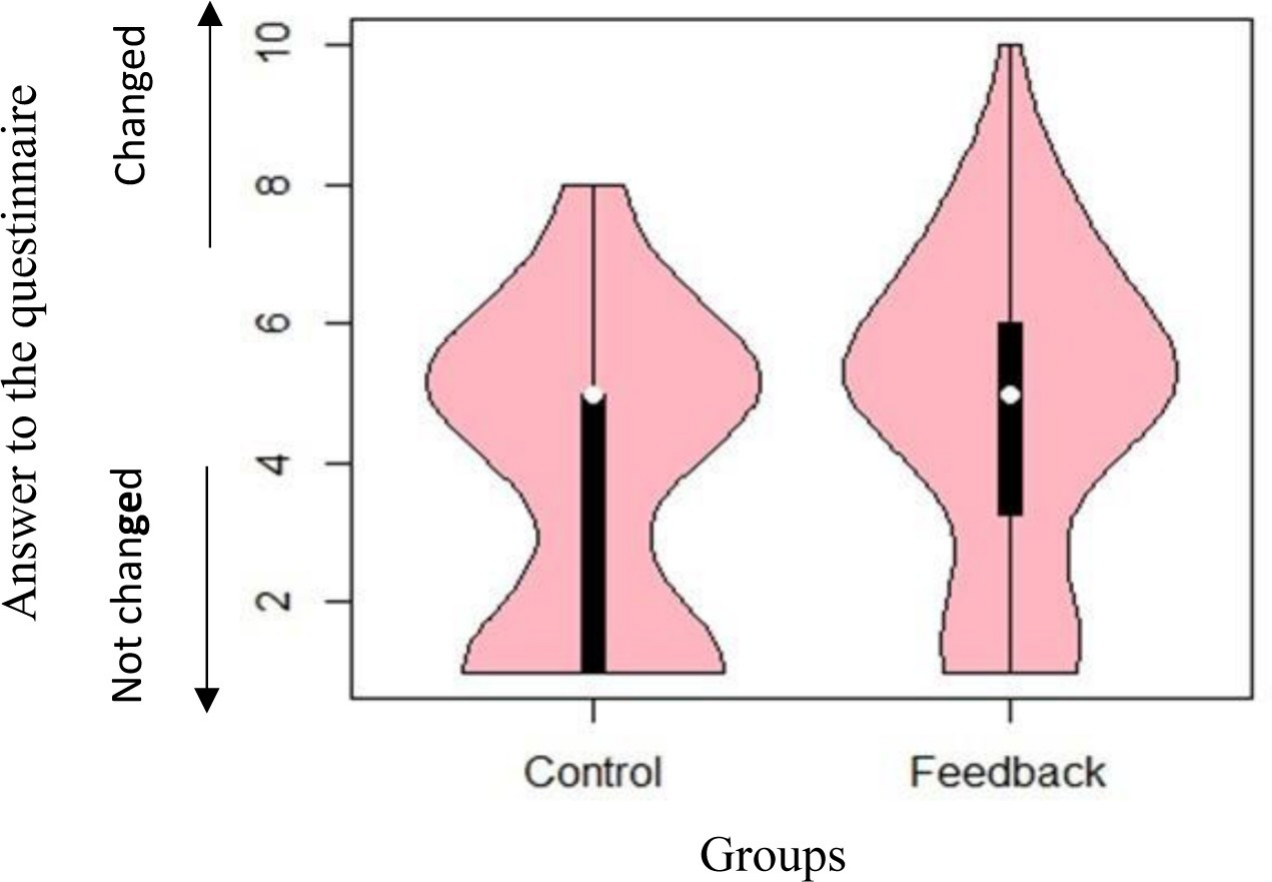
Distribution of responses to the question ‘According to you, by how much has your water usage changed?’.

Fig 8 shows the distribution of responses to the first question (i.e. ‘According to you, by how much has your water usage changed?’) using a violin plot. Because the distribution of responses to the question was not normally distributed, we conducted the Wilcoxon rank-sum test. It indicated that households in the feedback group felt that their water usage pattern had changed (*W* = 1580.00, *p* = 0.01).

**Fig 9:**
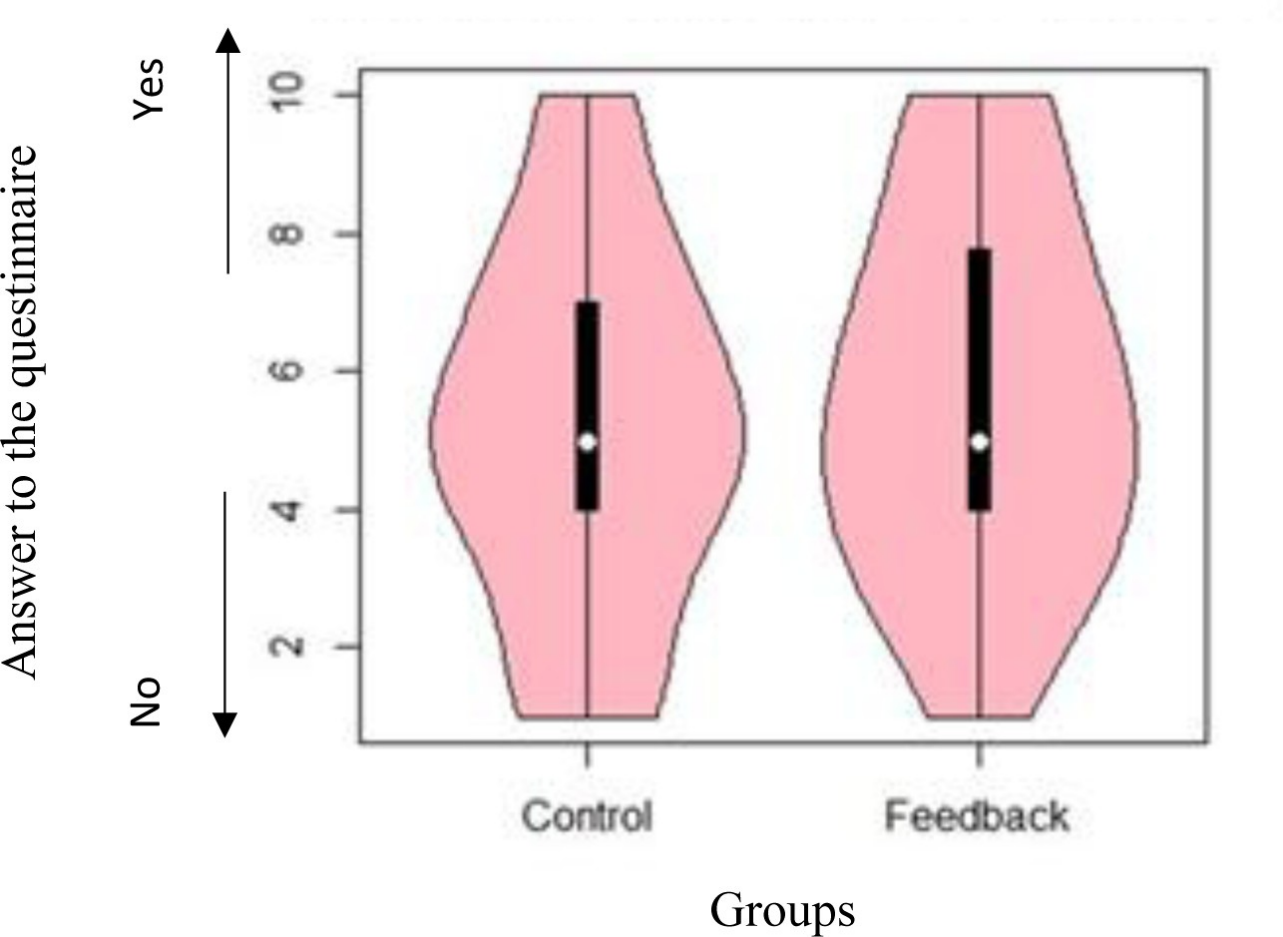
Distribution of responses to the question ‘Did you determine your water usage based on your own preferences?’

**Fig 10:**
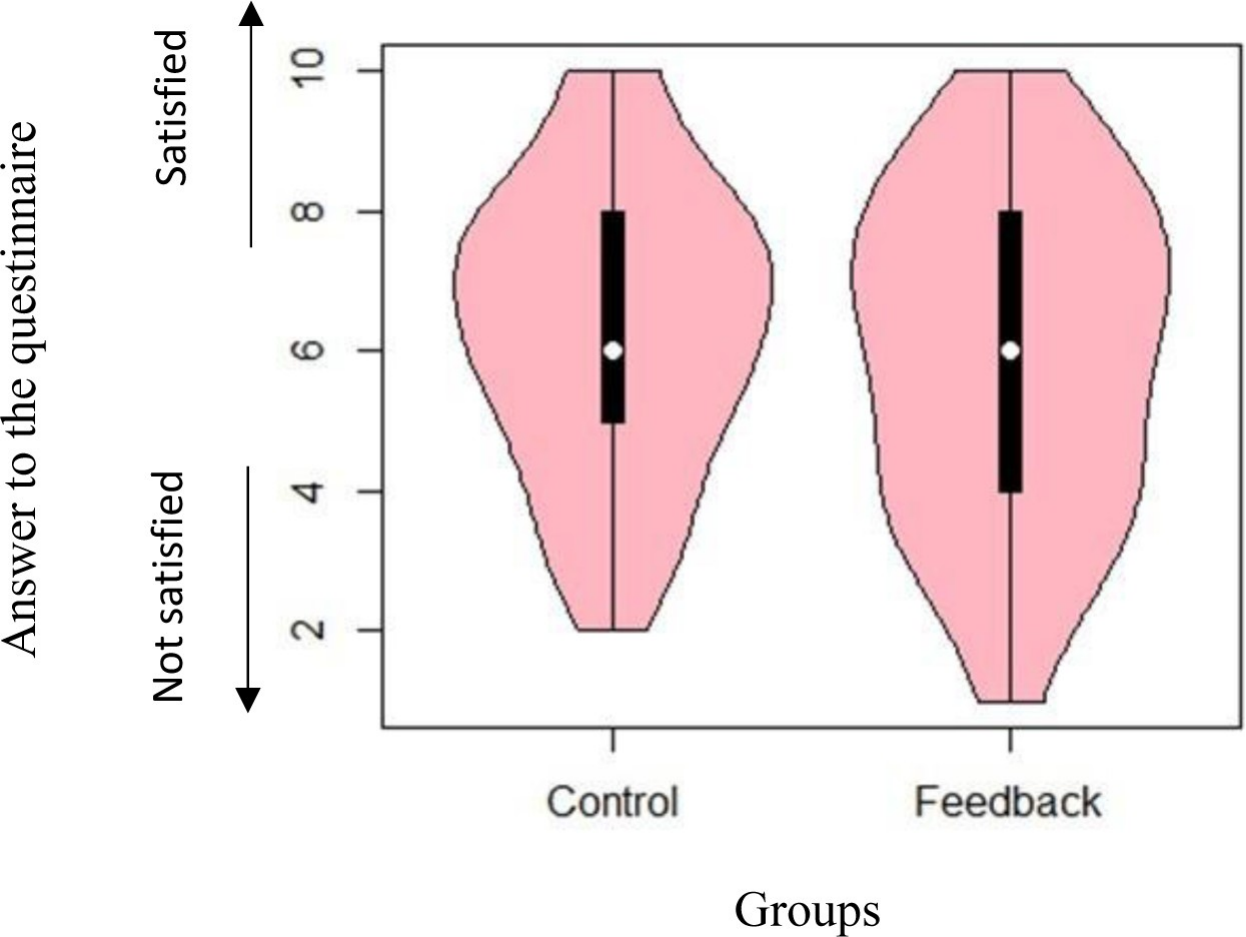
Distribution of responses to the question ‘Were you satisfied with your water usage?’

If such conscious change in water consumption and behaviour results in residents’ inconvenience or dissatisfaction, the use of these infographics may be problematic. However, concerning the responses to the second question (i.e. ‘Did you determine your water usage based on your own preferences?’), Fig 9 shows that there was no significant difference between the two groups (*W* = 1944.50; and *p* = 0.42). This result shows that people feel that they can freely determine their water use based on their preference. Concerning the responses to the third question (i.e.‘Were you satisfied with your water usage?’), Fig 10 shows that there was no significant difference between the two groups (*W* = 2304.5; and *p* = 0.38). The results show that people did not feel dissatisfied by seeing their water use in this context.

## 4. Discussion and conclusion

We presented visual feedback in the form of infographics representing water usage over six months to generate awareness that water resources are a public good. This resulted in water usage reduction for four months. The effect of the feedback was immediate and continuous during a certain period, however, it disappeared afterward. One possible reason was due to the lack of the long-term continuity of the effect, as shown by previous studies [31,32]. Another possibility was that water usage was reduced only during the summer, generally the period of perceived water shortage. Although there was no water shortage during the survey year, water shortages during the summer are a general concern for the Tokyo metropolitan area [33]. Some homemakers said that they increased their water-saving awareness because of the annual summer water shortages [34]. This means that the participants may have spent the summer months of the survey year anticipating a water shortage. However, this study alone could not confidently identify the reasons, and this is one of the study’s limitations. Further research could investigate whether this occurred because there were no longer water shortage concerns or because the effect had diminished over time.

Previous studies providing water usage feedback through social comparisons reported that only high water users reduced their water consumption and low water users showed a ‘boomerang effect’ [6,17, 35,36]. In contrast, as shown in Fig 6, there was no ‘boomerang effect’ evident in this study, and middle and low water users reduced consumption after seeing visual feedback.

Responses to the first question indicated that participants in the feedback group consciously changed their water usage behaviour. This is different from the results of previous studies, in which participants changed their behaviour instinctively [9]. Results from the responses to the second and third questions indicated no significant differences between the two groups regarding the freedom of choice and satisfaction about their water usage. Thus, the feedback from the infographic showed that people felt they determined their water usage based on their preference; in other words, it did not threaten participants’ free will, and they were satisfied with the amount of water usage of their household.

## Supporting information

S1 Fig Eight patterns of water vessel visuals with different water levels. (PDF)

S1 Table Means and 95% confidence intervals of LRP_n_. (PDF)

S2 Table Distribution of the numbers of family members. (PDF)

S3 Table Distribution of annual household income. (PDF)

S4 Table Result of difference-in-difference analysis with excluding outlier. (PDF)

S1 File (XLSX)

S2 File Full questionnaire. (PDF)
