# Supplementary figures and images for: Water demand management: Visualising a public good
Source: PLoS One. 2020 Jun 16;15(6):e0234621. doi: 10.1371/journal.pone.0234621 (PMC7297372; doi:10.1371/journal.pone.0234621)

Supplement 1 Eight patterns of water vessel visuals with different water levels

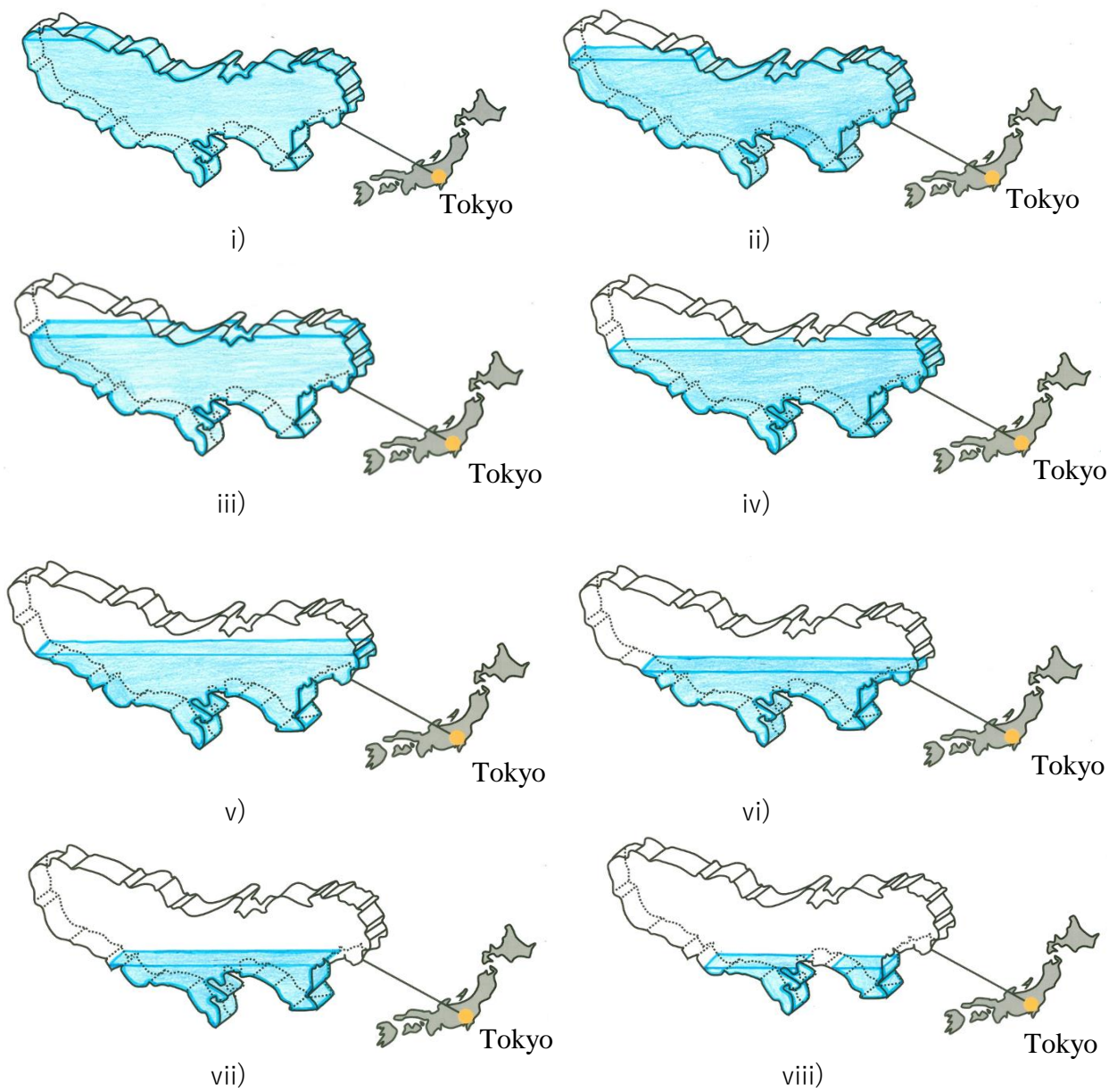

Supplement: S1 Fig — (PDF) [file pone.0234621.s001.pdf]
