# Supplementary material for: Water demand management: Visualising a public good
Source: PLoS One. 2020 Jun 16;15(6):e0234621. doi: 10.1371/journal.pone.0234621 (PMC7297372; doi:10.1371/journal.pone.0234621)
Supplement: S1 Table — (PDF) [file pone.0234621.s002.pdf]

Supplement 2 Means and 95% confidence intervals of LRP_n

	LRP_6	LRP_7	LRP_8	LRP_9	LRP_{10}
Control	0.015 (-0.012~0.042)	0.043 (0.008~0.078)	0.013 (-0.024~0.050)	0.002 (-0.028~0.033)	-0.046 (-0.090~-0.002)
Feedback	-0.021(-0.051~0.008)	-0.011(-0.057~0.035)	-0.041(-0.090~0.008)	-0.089(-0.051~0.003)	-0.010(-0.051~0.032)
