# Supplementary material for: Water demand management: Visualising a public good
Source: PLoS One. 2020 Jun 16;15(6):e0234621. doi: 10.1371/journal.pone.0234621 (PMC7297372; doi:10.1371/journal.pone.0234621)
Supplement: S2 Table — (PDF) [file pone.0234621.s003.pdf]

Suppliment 3 Distribution of the numbers of family members

						Number of households
Family size	1	2	3	4	5	6
Control Group	12	25	20	15	3	0
Feedback Group	11	21	8	15	3	1
