# Supplementary material for: Water demand management: Visualising a public good
Source: PLoS One. 2020 Jun 16;15(6):e0234621. doi: 10.1371/journal.pone.0234621 (PMC7297372; doi:10.1371/journal.pone.0234621)
Supplement: S3 Table — (PDF) [file pone.0234621.s004.pdf]

Supplement 4 Distribution of annual household income

Number of households

Annual Household Income	<4 Million JPY	4-6 Million JPY	6-8 Million JPY	>8 Million JPY
Control Group	16	11	16	32
Feedback Group	10	11	10	28
