# Supplementary material for: Water demand management: Visualising a public good
Source: PLoS One. 2020 Jun 16;15(6):e0234621. doi: 10.1371/journal.pone.0234621 (PMC7297372; doi:10.1371/journal.pone.0234621)
Supplement: S4 Table — (PDF) [file pone.0234621.s005.pdf]

Supplement 6 Result of difference-in-difference analysis with excluding outlier

Month	*t* value	*p* value
June	-1.808	0.072
July	-1.837	0.067
August	-2.320	0.021
September	-1.454	0.147
October	1.167	0.244
