# Supplementary material for: Water demand management: Visualising a public good
Source: PLoS One. 2020 Jun 16;15(6):e0234621. doi: 10.1371/journal.pone.0234621 (PMC7297372; doi:10.1371/journal.pone.0234621)
Supplement: S2 File — (PDF) [file pone.0234621.s007.pdf]

Supplement 5 Full questionnaire

1. By how much has your water usage changed?

Not changed ← → Changed

1	2	3	4	5	6	7	8	9	10

2. Did you determine your water usage based on your preferences?

No ← → Yes

1	2	3	4	5	6	7	8	9	10

3. Are you satisfied with your water usage?

Not satisfied ← → Satisfied

1	2	3	4	5	6	7	8	9	10
